# Mastery, physical activity and psychological distress in mid-aged adults

**DOI:** 10.1080/00049530.2022.2153623

**Published:** 2023-01-15

**Authors:** Adam J. Novic, Charrlotte Seib, Nicola W. Burton

**Affiliations:** a School of Applied Psychology, Griffith University, Brisbane, Australia; b Centre for Mental Health, Griffith University, Brisbane, Australia; c Menzies Health Institute Queensland, Griffith University, Gold Coast, Australia; d School of Nursing and Midwifery, Griffith University, Brisbane, Australia

**Keywords:** Exercise, mental health, well-being, self-control, mid-aged adults

## Abstract

**Objective:**

The objective was to investigate associations between mastery and physical activity with psychological distress in a population-based sample of mid-aged adults.

**Method:**

Self-reported measures of psychological distress, mastery and time spent in each of walking, moderate and vigorous physical activity in the previous week were examined in a cross-sectional sample of 7,146 adults aged 40–64 years (*M* = 53 years, *SD* = 6.5 years, 42.4% men). Generalized Linear Models were used to examine the inter-relationship between mastery and physical activity with psychological distress.

**Results:**

In fully adjusted models, only mastery was significantly associated with psychological distress (β = − 0.12, SE = 0.01, *p* < .01). There was no significant interaction between mastery and physical activity on psychological distress.

**Conclusions:**

Mastery may be an important resource against psychological distress. A sense of control may therefore be a key component for psychotherapeutic interventions to mitigate distress in mid-aged adults.

## KEY POINTS

What is already known about this topic:

1. Previous research indicates psychological distress is prevalent among Australian mid-aged adults.
2. Mastery and physical activity are resources shown to protect against psychological distress in mid-aged adults.
3. Longitudinal research with mid-aged adults has demonstrated a positive relationship between mastery and physical activity.

**What this topic adds:**

1. The current study showed higher mastery was associated with lower psychological distress in a sample of mid-aged adults.
2. No relationship was observed between physical activity and distress or for an interaction between physical activity and mastery.
3. This evidence may inform the development of interventions to mitigate distress in mid-aged adults.

Psychological distress is associated with significant morbidity and premature death globally. Within Australia, approximately 2.4 million adults (i.e., 13%) experience high/very high psychological distress each year with mid-aged adults (45 to 64 years) the highest proportion of all age groups reporting very high levels of psychological distress (Australian Bureau of Statistics, 2018). Psychological distress increases risk of major chronic diseases such as arthritis, cardiovascular disease, chronic obstructive pulmonary disorder, and has a positive dose-response association with all-cause mortality (McLachlan & Gale, 2018; Russ et al., 2012). Psychological distress causes significant economic burden and is estimated to cost AUD$5.9 billion in relation to reduced productivity and increased disability support, even after accounting for diagnosed mental and somatic illness (Hilton et al., 2010; Rai et al., 2012). More research is needed therefore, to identify modifiable factors associated with distress, to inform strategies to reduce this burden.

Mastery is a predictor of emotional well-being in mid-aged adults and can be an important psychological resource to protect against distress (Windsor & Anstey, 2010). Mastery is a malleable self-concept central to managing existing stressors and is typically conceptualised as a sense of control – as opposed to fatalistic rule – that one has over the important forces that affect one’s life (Pearlin & Schooler, 1978). Mastery has an inverse association with negative affect and a positive association with life satisfaction and positive affect (Windsor et al., 2009). For people in early mid-age adulthood, mastery has been shown to have a moderate negative correlation with anxiety (*r* = ‒0.43, *p* < .01) and depression (*r* = ‒0.51, *p* < .01) (Burns et al., 2011).

Physical activity can also protect against psychological strains and stressors. People that are more frequently physically activity are less likely to experience psychological distress than those who do no or little (i.e., < 1/week) physical activity (Hamer et al., 2009; Perales et al., 2014). All forms of physical activity, whether light, moderate or vigorous have been shown to have long-term protective effects against psychological distress (Sheikh et al., 2018). For those over the age of 45 years, engaging in physical activity has been shown to reduce the odds of psychological distress (Plotnikoff et al., 2015). Meta analyses of prospective studies have shown that physical activity is protective against incident depression (adjusted OR = 0.78, 95%CI: 0.70 to 0.87) and anxiety (adjusted OR = 0.66, 95% CI = 0.53–0.82) in adults (McDowell et al., 2019; Schuch et al., 2018).

There may be an interrelationship between mastery and physical activity. Research has explored the mediating role of both mastery and physical activity on distress. Longitudinal research with an Australian sample of adults (n = 7,485) indicated a positive association between mastery and physical activity, which in turn was associated with better physical and psychological health (Sargent-Cox et al., 2015). A greater sense of mastery may, therefore, support adults to be physically active and result in reductions in distress. Mastery has also been hypothesised as a mechanism by which physical activity positively impacts on mental health (Biddle, 1993). Mastery has also been shown as a mediator of the relationship between physical activity and psychological distress in people aged 20 to 65 years, as well as a moderator of the relationship with inactive individuals reporting low mastery experiencing the highest level of distress (Martin & Wade, 2000). However, among people aged over 65 years, no association was demonstrated between mastery and distress in inactive individuals reporting high distress (Cairney et al., 2005). This suggests that an inter-relationship between mastery and physical activity may be less salient among older adults and prompts further research with other age groups such as mid-aged adults.

Although mastery and physical activity have separately been shown to be inversely associated with psychological distress, and have also been positively associated with each other, studies have not investigated these variables concurrently as independent predictors of distress, or their possible interaction in mid-aged adults. This study aimed to explore cross-sectional evidence for the inter-relationship between mastery and physical activity with psychological distress in mid-aged adults.

## Methods

This study presents a secondary data analysis of the HABITAT study, a multilevel longitudinal cohort study investigating physical activity, as well as potentially related psychological, social and environmental factors in mid-aged and older men and women living in Brisbane, Australia (Burton et al., 2009). The HABITAT survey was awarded initial ethical clearance by the QUT Human Research Ethics Committee in 2007 (ID3967 H).

The design and recruitment process have been described in detail elsewhere (Burton et al., 2009). Briefly, a multi-stage probability sampling design, which was used to obtain a stratified random sample of 200 neighbourhoods from Census Collection Districts (CCDs) ranked by the Index of Relative Socioeconomic Disadvantage, a score used to reflect attributes such as proportion of low-income families and individuals with low educational attainment and workers in relatively unskilled occupations. From each of the 200 neighbourhoods, an average of 85 households with at least one person aged 40 to 65 years (as of March 2007) was identified using data from the Australian Electoral Commission on people’s names, address, and date of birth. The final stage of the sampling procedure involved randomly selecting one person aged 40 to 65 years from each selected household (i.e., 17,000 mid-aged adults) who were then invited to participate by mail.

Data were collected using structured self-completed mail questionnaires which were tailored to the local area and age of the cohort. Participants were mailed advanced notice of survey delivery and reminders/thank you for completion and return. People who did not respond were sent a replacement questionnaire after seven weeks of the initial mailout. Each person was assigned a unique identification code which was printed on their questionnaire to enable matching across survey waves. Overall, the HABITAT survey included five waves across a nine-year period, the first wave in 2007 (n = 11,035, 68.43% response rate) and the last in 2016 (n = 5,187, 58.77% response rate). When compared with 2006 census data, the 2007 HABITAT sample was broadly representative of the wider Australian mid-aged population (Turrell et al., 2010).

### Study sample

**Figure 1.**
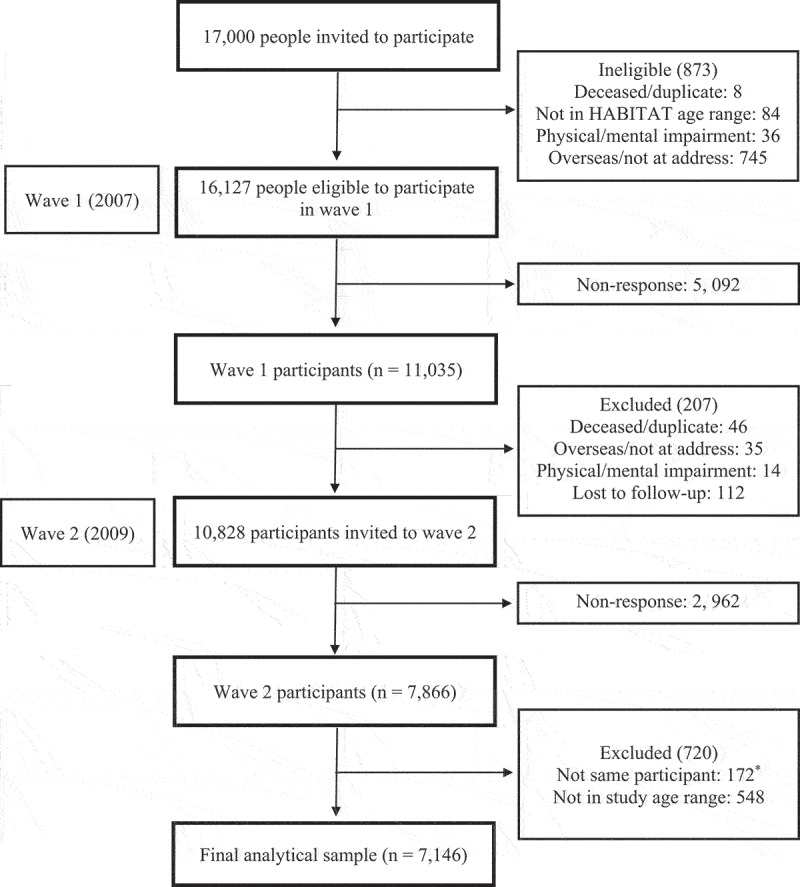
Flowchart of derived analytic sample.

This analysis used 2009 HABITAT survey data as it included measures of psychological distress, mastery, and physical activity. Only those respondents aged 40 to 64 years in 2009 were considered for the current study given the study focus on mid-aged adults. Socio-demographic data on country of birth and highest completed educational qualification were obtained from the 2007 baseline survey as these were not collected in 2009. The analytical sample comprised 7,866 participants (72.65% response rate) from 10,828 mid-aged adults eligible to participate in wave 2 of the HABITAT study. Longitudinal inconsistencies in the dataset (e.g., changes in gender from baseline) were reviewed with 172 cases excluded due to suspected change in respondent across waves. The analysis comprised a final sample of 7,146 adults aged between 40 and 64 years. The process for deriving the final sample is shown in Figure 1.

*Longitudinal inconsistencies in the dataset (e.g., changes in gender) were assessed. Only those that were indicated to represent a single respondent across waves were included in analyses.

### Measures

The primary outcome was measured using the abbreviated Kessler 6 (K6) scale (Kessler et al., 2002). This six-item instrument assesses how often respondents experienced anxio-depressive symptoms in the past month on a 5-point Likert scale ranging from 0 *“none of the time”* to 4 *“all of the time”*. Items are summed resulting in a total score ranging from 0 to 24, with higher scores indicating more psychological distress. Previous work has classified scores of 5–12 as moderate distress, and a score ≥13 as severe distress (Prochaska et al., 2012). The K6 had acceptable internal consistency in the analytic sample of the current study (Cronbach’s *α* = 0.87).

Mastery was assessed using the Pearlin Mastery Scale (PMS) (Pearlin & Schooler, 1978), a 7-item scale that measures the extent to which an individual regards aspects of life as being under personal control or fatalistically ruled. Respondents are asked to indicate to what extent they agree with items using a 5-point Likert scale ranging from 1 *“strongly disagree*” to 5 *“strongly agree”* and items are summed resulting in a total score ranging from 7 to 35 with higher scores indicating a greater sense of mastery. The PMS had a satisfactory internal consistency in the analytic sample of the current study (Cronbach’s *α* = 0.81).

Physical activity was assessed using items from the Active Australia Survey (AAS) (Australian Institute of Health and Welfare, 2003) for participation in walking, moderate physical activity (e.g., gentle swimming, social tennis, golf), and vigorous physical activity (e.g., jogging, aerobics, competitive tennis), excluding household and gardening activity. Respondents report the duration of each category of physical activity in the last week. The AAS items exhibit good reliability and acceptable validity (Australian Institute of Health and Welfare, 2003; Brown et al., 2002) and have been recommended as suitable for use in Australian population-based research (Brown et al., 2004). Total time in physical activity was a weighted calculation computed by adding time spent in walking, moderate physical activity, and two times the amount spent in vigorous physical activity given the higher intensity (Australian Institute of Health and Welfare, 2003). Physical activity was truncated according to Active Australia Survey guidelines to minimise errors due to over-reporting (Australian Institute of Health and Welfare, 2003).

Several covariates identified from previous research as potential confounders with psychological distress (Enticott et al., 2016; McLachlan & Gale, 2018; Pearlin et al., 1981) were also considered in this analysis. These were self-reported and included demographic variables (age, gender, country of birth, and highest educational qualification) and general health (rated as poor, fair, good, very good, or excellent). To avoid considerable discrepancy in group frequencies, three general health response groups were created by collapsing poor and fair responses, and very good and excellent responses.

### Statistical analyses

All data were analysed using the Statistical Package for the Social Science (SPSS) version 26 (IBM Corp, 2019). The distributions of key variables (sociodemographic variables, mastery, time in physical activity, psychological distress) were graphically inspected using frequency distributions and normal P-P plots and were summarised as means (standard deviations), medians (interquartile range [IQR]), and proportions (number, percentage). Bivariate associations between the key (mastery, physical activity) and sociodemographic (gender, employment status, education, health, country of birth) study variables and the outcome of distress were estimated using correlation coefficient for continuous data and effect sizes derived from parametric (independent samples t-tests and analysis of variance) and non-parametric (Mann Whitney-U and Kruskal Wallis-H) methods, where appropriate. Bivariate analyses were conducted to explore multicollinearity model assumptions among key predictor variables and to determine inclusion in multivariable modelling, with an alpha level of .05 required.

Model assumptions were tested before undertaking multivariate analyses. Normality assumption for ordinary least squares (OLS) regression was assessed by examining the regression standardised residual, resulting in no transformations. To assess for multicollinearity among model predictors, variance inflation factors (VIF) were estimated with the highest VIF value among predictors being 1.18 (health rating). A scatter plot of the regression standardised residual against the regression standardised predicted value indicated violation of homoscedasticity; therefore, a generalised model was preferred to accommodate the nature of the data.

Inspection of the Normal P-P plot suggests the outcome variable was best represented by a gamma distribution. A mixed effect model was considered to accommodate for non-independence of observations through cluster random effects (i.e., neighbourhood based on CCDs). Appropriateness of this type of modelling was determined by evaluating the intraclass correlation (ICC) statistic and design effect statistic based on the simplest intercept-only (i.e., null) model (Hox et al., 2017). The obtained ICC was based on a gamma distribution with log link and indicated that only 3.03% of the variation in psychological distress scores lay between neighbourhoods, suggesting limited influence of clustering given a value less than 5% (Heck et al., 2014; Nakagawa et al., 2017). Based on an average of 11.79 observations in each cluster, the computed design effect was 1.33. With an average cluster size over 10, a design effect less than 2 is suggested to limit negative bias of standard errors as a result of nested data and thus providing further evidence for independence of the data (Lai & Kwok, 2015; Muthen & Satorra, 1995). A single-level model was preferred for parsimony given the near independence of observations and model variables structured on the same level. The independent and combined contribution of mastery and time spent in physical activity (weighted) in predicting psychological distress were assessed by examining main and interactive effects using a Generalized Linear Model (GzLM) to account for observed heteroscedasticity.

A stepped model approach was used in the analyses. Three models were derived to examine the influence of included covariates on model coefficients. The first model included only the key predictor variables of physical activity and mastery. The second model added sociodemographic covariates in addition to variables included in the first model. The final model added the self-reported health covariate to variables in the second model.

## Results

### Missing data analysis

Missing data analyses were performed identifying data to be missing completely at random (Little’s MCAR, χ^2^(15115) = 11956.06, *p* = 1.00). Overall, there was only a small amount of missing data on variables: age and gender (0%), country of birth (0.4%), highest education achieved (0.3%), health rating (2.6%), time spent in vigorous intensity physical activity (3.2%), time spent in moderate intensity physical activity (4.0%), time spent walking (3.4%), mastery (2.4%), and psychological distress (2.3%). As such, missing data for time spent in physical activity was then managed according to published protocols and guidelines (Australian Institute of Health and Welfare, 2003). Where participants did not report time spent in either one or two physical activity intensities, these were replaced with a zero (0) value and cases where no physical activity data was reported (2.5%) were excluded from analyses. Cases with missing data on remaining variables were excluded from analyses.

### Main analysis

Summary characteristics of the study sample are displayed in Table 1. The average age of a participants was 53 years (*SD* = 6.50), and most were female (57.6%), born in Australia (76.6%), in the paid workforce (77.1%), and residing with a partner (67.9%). Respondents generally reported low levels of psychological distress (median = 2, IQR: 1–5) and high levels of mastery (median = 27, IQR: 24–29). The median total time spent in physical activity was 240 weighted minutes/week (IQR: 90–550).

**Table 1.** Summary characteristics of analytic sample of adults aged 40 to 64 years (n = 7,146).

	n (%)^a^
Gender	
Men	3,033 (42.4)
Women	4,113 (57.6)
Country of Birth	
Australia	5,449 (76.3)
Other	1,666 (23.3)
Education	
School (year 12)	2,655 (37.2)
Certificate/Diploma	2,076 (29.1)
Bachelor/Postgraduate Degree	2,397 (33.5)
Employment Status	
In paid workforce	5,054 (70.7)
Not in paid workforce	1,497 (20.9)
Living Arrangement	
Living alone	1,064 (14.9)
Single parent with 1+ children	474 (6.6)
Single with friends/relatives	315 (4.4)
Couple with no children	1,979 (27.7)
Couple with 1+ children	2,873 (40.2)
Other^b^	176 (2.5)
Reported Health Rating	
Fair	1,224 (17.1)
Good	2,790 (39.0)
Excellent	2,945 (41.2)

^a^total may differ due to missing data.

^b^Alternate living arrangement (e.g., couple residing with nephew).

Bivariate analyses showed significant negative associations between psychological distress and mastery (*r*_*s*_(6918) = −.53, *p* < .01), and time spent in physical activity (*r*_*s*_(6820) = −.08, *p* < .01), while positive associations were found between mastery and time spent in physical activity (*r*_*s*_(6813) = .13, *p* < .01). Bivariate analyses of grouped data are presented in Table 2.

**Table 2.** Bivariable associations of grouped data between psychological distress^a^, mastery^b^ and physical activity^c^.

	Psychological distress	Mastery	Physical activity
	Median (IQR)	Mean (SD)	Median (IQR)
Gender			
Men	2 (0–4)*	26.82 (4.51)*	300 (90–600)**
Women	2 (1–5)	26.53 (4.58)	240 (90–480)
Country of Birth			
Australia	2 (1–5)	26.68 (4.58)	240 (90–563)
Other	2 (0–5)	26.58 (4.45)	240 (80–530)
Education			
School (year 12)	2 (0–5)*	26.01 (4.65)**	210 (60–480)**
Certificate/Diploma	2 (1–4)	26.73 (4.36)	270 (90–600)
Bachelor/Postgraduate Degree	2 (1–4)	27.31 (4.52)	300 (120–600)
Employment Status			
In paid workforce	2 (1–4)**	26.90 (4.40)**	240 (90–540)
Not in paid workforce	2 (1–5)	25.83 (4.97)	240 (90–583)
Living Arrangement			
Living alone	2 (1–6)**	25.98 (5.02)**	240 (70–540)**
Single parent with 1+ children	3 (1–6)	25.81 (4.77)	210 (80–480)
Single with friends/relatives	3 (1–5)	25.83 (4.60)	180 (73–435)
Couple with no children	2 (0–4)	27.09 (4.36)	260 (90–600)
Couple with 1+ children	2 (1–4)	26.94 (4.34)	270 (90–555)
Other^d^	3 (1–7)	24.90 (5.30)	180 (60–450)
General Health Rating			
Fair/poor	4 (2–8)**	24.16 (4.90)**	120 (30–300)**
Good	2 (1–5)	26.14 (4.22)	230 (80–480)
Very good/Excellent	2 (0–3)	28.14 (4.16)	360 (135–720)

^a^Psychological distress was measured using the Kessler 6 (K6) scale (Kessler et al., 2002) ranging from 0–24; ^b^Mastery was measured using Pearlin Mastery Scale (PMS) (Pearlin & Schooler, 1978) ranging from 7–35; ^c^Physical activity was measured using the Active Australia Survey (AAS) (Australian Institute of Health and Welfare, 2003) and is reported as weighted mins per week. ^d^Alternate living arrangement (e.g., couple residing with nephew). The sample size was n = 7,146.

* *p* < .05 ** *p* < .01.

Regarding the primary outcome, results indicated greater psychological distress among women (*U* = 5753504.00, *p* = .02, Cohen’s *d* = 0.06), those not in the paid workforce (*U* = 3401645.50, *p* < .01, *d* = 0.09), those with school only level education (*χ*^*2*^(2) = 6.40, *p* = .04, *η*^*2*^ <0.01), and those reporting poor/fair general health (*χ*^*2*^(2) = 558.94, *p* < .01, *η*^*2*^ = 0.08). Country of birth was not shown to be associated with psychological distress in this sample (*U* = 4256105.50, *p* = .24).

Table 3 presents the multivariate analysis. Psychological distress was modelled as a function of mastery and physical activity using a Gamma GzLM with a log link function (to ensure positive fitted values). Models were run with and without covariates to determine the influence of confounding factors on parameter estimates. An inverse relationship was noted between psychological distress and mastery (β = −0.13, SE = 0.01, *p* < .01) which was unchanged following adjustment for socio-demographic characteristics (Model 2) and self-rated health status (Model 3). The fit statistics were compared, with the final adjusted model a good fit for the data (χ^2^(6565) = 6662.18, *p* = .20).

**Table 3.** Generalised linear models of mastery and physical activity adjusted for sociodemographic and health characteristics.

Variables	Model 1 β (SE)	Model 2 β (SE)	Model 3 β (SE)
Mastery x Time in Physical Activity	0.00 (0.00)	0.00 (0.00)	0.00 (0.00)
Mastery	−0.13 (0.01)**	−0.13 (0.01)**	−0.12 (0.01)**
Time in Physical Activity	0.00 (0.00)	0.00 (0.00)	0.00 (0.00)
Age	-	−0.02 (0.00)**	−0.02 (0.00)**
Gender			
Men	-	Ref	Ref
Women	-	0.05 (0.04)	0.07 (0.04)
Country of Birth			
Australia	-	Ref	Ref
Other	-	−0.01 (0.05)	−0.01 (0.05)
Education			
School	-	Ref	Ref
Certificate/Diploma	-	0.03 (0.05)	0.05 (0.05)
Bachelor/Postgraduate	-	0.09 (0.05)	0.13 (0.05)*
General Health			
Fair/Poor	-	-	Ref
Good	-	-	−0.25 (0.06)**
Very Good/Excellent	-	-	−0.46 (0.06)**
AIC	19986.24	19877.78	19331.84
BIC	20020.33	19945.91	19413.33

Model 1, unadjusted model; Model 2, adjusted for age, gender, country of birth, education; Model 3, adjusted for model 2 and general health. The sample size was n = 7,146.

* *p* < .05 ** *p* < .01.

## Discussion

Identifying potentially modifiable factors to mitigate distress can inform suitable interventions to reduce associated burden. This study sought to assess the inter-relationships of mastery and physical activity on psychological distress in mid-aged adults. After adjusting for multiple potential confounders, our data indicated that mastery had a significant inverse association with psychological distress in mid-aged adults. In both adjusted and unadjusted models, there was no evidence for the combined effect of mastery and physical activity on distress in the sample.

Mastery was shown to have a strong negative association with psychological distress in mid-aged adults and might therefore be a key resource to protect against distress. This result is consistent with previous longitudinal research suggesting increases in mastery over time are associated with corresponding increases in positive affect in mid-aged adults (Windsor & Anstey, 2010). Mastery is considered a part of one’s self-concept central to managing stressors and can help regulate experiences of psychological distress (Pearlin & Schooler, 1978). Research has also suggested mastery is one mechanism by which exercise has a positive impact on psychological distress (Biddle, 1993; Martin & Wade, 2000).

Contrary to previous findings (Plotnikoff et al., 2015; Schuch et al., 2018), although our results demonstrated a significant bivariate association between physical activity and distress, they did not show the amount of time spent in physical activity to explain scores on psychological distress. This may reflect differences in the measurement of physical activity as previous studies which have demonstrated a relationship have assessed *frequency* of physical activity (Hamer et al., 2009; Perales et al., 2014) while our study assessed time. A review of studies on physical activity and anxiety also demonstrated mixed evidence of an association across studies with different measures of physical activity (e.g., frequency, intensity, time categories, meeting physical activity recommendations) (McDowell et al., 2019). Results may also reflect a bidirectional relationship between physical activity and distress as previous research has observed a reciprocal relationship over time (Gucciardi et al., 2020). Previous studies exploring the relationship between physical activity and distress have been conducted with samples across broad age ranges, including younger and older adults (Hamer et al., 2009; Sheikh et al., 2018), have shown significant associations with weekly engagement in, and intensity of, physical activity and age. Differences in physical activity among people across the adult lifespan may potentially influence psychological outcomes for different age groups (McDowell et al., 2019). Therefore, other factors (e.g., general health) may be more important influences on distress for mid-aged adults.

There was little evidence of an interrelationship between mastery and physical activity on psychological distress in the current study. Given the reported high levels of mastery in our study participants, this may suggest that there is no further protective effect against distress from physical activity. Notably, our study did find a positive relationship between mastery and time spent in physical activity, suggesting that these two factors may have a bidirectional relationship. Previous research suggests that higher mastery is positively associated with higher physical activity levels, which in turn is associated with better physical and psychological health (Sargent-Cox et al., 2015). Another consideration is the frequency and intensity “dose” of physical activity, which has also emerged as an additional consideration when examining the relationship with psychological distress (Perales et al., 2014). The influence of dose of physical activity on mastery and distress may provide further insights for development of interventions to mitigate psychological distress for mid-aged adults.

Whilst our study offers important insights, some methodological limitations should be acknowledged. The cross-sectional design limits inferences about the direction of observed relationships. It may be that distress constrains participation in physical activity and lowers levels of mastery in mid-aged adults. The survey method (self-report data) did not allow for collection of clinical indicators of health (e.g., blood pressure, BMI) and may introduce a social desirability bias such that respondents answered questions about key variables in a manner that would be viewed as favourable. Among our participants, two-thirds were meeting current physical activity guidelines of at least 150 mins/week (World Health Organization, 2010), approximately half reported mastery in the top quartile of scores, and three quarters had minimal levels of distress. The limited variability on the distress outcome may have constrained evidence of inter relationships. However, the self-report measures used in the study are well established and are widely used (Brown et al., 2004; Kessler et al., 2002; Pearlin et al., 1981) with generalisability aided by use of a multi-stage probability sampling design. The large sample size also allowed us to explore a range of possible covariates in the analyses.

The results of our study highlight the potential importance of mastery as a protective psychological resource against distress. For mid-aged adults, a sense of psychological control appears to relate to psychological health more so than physical activity and could be included as a key component in psychotherapeutic interventions to mitigate distress. Contemporary approaches, such as Acceptance and Commitment Therapy (ACT) may promote mastery through, for example, principles related to present-moment awareness (Pagnini et al., 2016), engaging in meaningful activities (Reich & Zautra, 1990), and defusion and cognitive flexibility (Hayes et al., 2011). Future work could evaluate the impact of a psychotherapeutic intervention promoting mastery to reduce psychological distress in mid-aged adults.

In conclusion, this study of mid-aged Australian adults suggests that mastery is inversely associated with psychological distress. Future research is needed to explore the longitudinal associations between mastery, dose of physical activity, and distress, as well as underlying mechanisms. Understanding the role of psychological and physical resources, such as mastery and physical activity, in mid-aged adults may provide a means to manage distress, and reduce associated morbidity, premature death and economic burden in this population.

## Data Availability

The data that support the findings of this study are not publicly available but are available on reasonable written request: a written request can be submitted to HABITAT lead investigator, Professor Gavin Turrell (gavin.turrell@rmit.edu.au).
